# Knowledge, attitude and practices of residents toward antimicrobial usage and resistance in Gondar, Northwest Ethiopia

**DOI:** 10.1186/s42522-022-00066-x

**Published:** 2022-05-18

**Authors:** Haileyesus Dejene, Rediet Birhanu, Zewdu Seyoum Tarekegn

**Affiliations:** 1 Department of Veterinary Epidemiology and Public Health, College of Veterinary Medicine and Animal Sciences, University of Gondar, Gondar, Amhara Ethiopia; 2 Department of Veterinary Paraclinical Studies, College of Veterinary Medicine and Animal Sciences, University of Gondar, Gondar, Amhara Ethiopia

**Keywords:** Antimicrobial use, Knowledge, Attitude, Practice, Resistance, Gondar, Ethiopia

## Abstract

**Background:**

Antimicrobials are essential for human and animal health. Drug resistance to an antimicrobial agent follows the introduction of a new antimicrobial agent. Evidence suggests that the public plays an important role in the risk, increase, and spread of antimicrobial resistance. This study aimed to assess the knowledge, attitudes, and practices of the Gondar City residents regarding antimicrobial use and resistance.

**Methods:**

A cross-sectional study was conducted from April to July 2021 on 400 randomly selected Gondar city residents using a pretested semi-structured questionnaire. The descriptive and chi-square tests were used to analyse the data.

**Results:**

The response rate was one hundred percent. Approximately 75% of respondents were men, with 32% having completed secondary school. Nearly 74% and 35% of participants were married and worked in various government jobs. Furthermore, 48%, 54%, and 50% of respondents, respectively, had moderate knowledge, a positive attitude, and good practice concerning antimicrobial use and resistance. The chi-square analysis revealed a significant (*p* < 0.05) disparity between knowledge and educational level, marital status, and position in the house. The respondents’ attitude levels were also significantly associated (*p* < 0.05) with their educational level, marital status, occupation, and position in the house. Respondents’ practice levels were also significantly associated (*p* < 0.05) with their educational level and occupation. The study also found a significant relationship between respondents’ knowledge and attitude (χ2 = 215.23, *p* ≤ 0.01), knowledge and practice (χ2 = 147.2, *p* ≤ 0.01), and attitude and practice (χ2 = 116.03, *p* ≤ 0.01).

**Conclusion:**

This study found that study participants had some misconceptions about antimicrobial use and resistance. As a result, both enforcing antimicrobial regulation and educating people about antimicrobial use are advised.

**Supplementary Information:**

The online version contains supplementary material available at 10.1186/s42522-022-00066-x.

## Introduction

Microbes have been around for a million years and are one of the oldest creatures on the planet. While other advanced, massive ancient animals and plants perished, these tiny microorganisms adapted, developed, and survived throughout the eras. On the other hand, microbes are thought to be some of nature’s most adaptable and successive creatures. Since that time, these microbes have been subjected to antibiotics derived from other microorganisms, such as *Penicillium notatum* [1]. Furthermore, hundreds of natural, semi-synthetic, and synthetic antimicrobial molecules are used to treat infections in both humans and animals [2]. These antimicrobials have been widely used in animals for disease prevention, control, and treatment, as well as growth promoters. Antimicrobial drug use has become widespread over several decades, and these drugs have been widely misapplied in both humans and food-producing animals in ways that promote the selection and spread of resistant microbes [3].

Misuse and overuse of various antimicrobial agents in the health care setting and the agricultural industry are regarded as major contributors to the emergence of antimicrobial resistance. Besides that, spontaneous evolution, pathogen mutation, and the transmission of resistant genes via horizontal gene transfer are significant contributors to antimicrobial resistance [4]. More than half of all medicines are prescribed, dispensed, or sold improperly and do not follow the principle of rational drug use, and more than half of patients fail to take them correctly. This erroneous dose leads to an increase in antimicrobial resistance. Despite ongoing efforts to improve antimicrobial prescribing and address issues such as self-prescribing, unnecessary use for viral infections, dosing errors, and excessive treatment durations, the global rate of antimicrobial-resistant infections is increasing [5].

Antimicrobial resistance and the rise in MDROs globally are associated with increased morbidity and mortality, cross-transmission within and between healthcare settings, and increased consumption of limited patient-care resources. Despite increased awareness, the publication of antimicrobial stewardship guidelines, and a variety of initiatives, the proportion of resistant strains causing both health-care and community-associated infections continues to rise, while the number of new antimicrobials continues to fall [6].

In general, antimicrobial resistance is a global public health concern that has been exacerbated by the overuse of antimicrobials around the world [7]. Further to that, it is a major concern for most African countries with low and middle incomes, which are associated with poverty, a high prevalence of infectious diseases, and uncontrolled antimicrobial use in animals and humans [8, 9]. There is little information available about antimicrobial use and resistance in Ethiopia. Similarly, some studies show that inappropriate antibiotic use is linked to a variety of factors such as a low education level, job engagement, and a lack of knowledge about the use of human antibiotic preparations to treat animals [10, 11]. However, it is questionable to what extent knowledge, attitudes, and behavioural practices regarding animal and human antimicrobial usage and resistance are in the study area. Thus, the study of human and animal antimicrobial usage and resistance in the study area contributes to the condition by identifying the factors that contribute to a low level of knowledge, attitudes, and behavioural practices, thus allowing the community to be aware of the condition and apply possible intervention measures to reduce the risks. As a result, the purpose of this study was to assess residents’ knowledge, attitudes, and behavioural practices regarding antimicrobial usage and resistance in Gondar, northwest Ethiopia.

## Material and methods

### Study area

The study was conducted in Gondar, a city in northwest Ethiopia, 740 kilometres from the capital, Addis Ababa. The city is a historic and tourist destination in the country. The city's elevation ranges from 1800 to 2200 meters above sea level. It receives 1000 millimetres of rain per year on average. The city’s annual maximum and minimum temperatures are 30.7°C and 22°C, respectively, with an overall average temperature of 26°C. The relative humidity in the city ranges from 60% to 70% during the rainy season and from 30% to 40% during the dry season. Gondar had a population of 500,788, with 300,000 men and 200,788 women [12].

### Study design

From April to July 2021, a cross-sectional study was conducted in Gondar, Northwest Ethiopia, to assess residents’ knowledge, attitude, and behavioural practices regarding human and animal antimicrobial use and resistance. The study was conducted following the Helsinki Declaration as well as national and institutional standards.

### Study population and sample size

The source population consisted of adults of both genders who were at least 18 years old and lived in Gondar city. Thrusfield’s formula for a single population was used to determine the number of samples included in this study [13]. The 95% confidence interval, 5% marginal error, and 50% for antimicrobial use during the past twelve months were considered to calculate the sample size. As a result, 384 people from Gondar city were included in the study. However, after accounting for a 5% non-response rate, the final sample size was 400. To select study participants, a random sampling method with a lottery system was used.

### Data collection and tools

#### Data collection

For data collection via interview, a semi-structured questionnaire was used. The questionnaire was developed after reviewing the literature on how to conduct a KAP survey as well as global antimicrobial studies. The questionnaire consisted of four sections, with the majority of the questions being closed-ended. Two epidemiology and public health experts reviewed the questionnaire items for content validity. Based on expert feedback and recommendations, questionnaire items were modified to better suit the local population. Furthermore, before data collection, participants in this study were given verbal information to inform them of the purpose of the study, and they were free to leave the interview at any time. Furthermore, all the data will be kept securely.

#### Measurement tools

##### Antimicrobial usage and resistance knowledge

Nine questions were asked to determine the residents’ level of knowledge concerning antimicrobial use and resistance. Six of the questions were on a Likert scale, while the other three were open-ended, allowing respondents to express their opinions. The answers to closed-ended questions were scored as 3 (correct answer), 2 (uncertain answer), and 1 (no answer or for a wrong answer). The scores ranged from 6 to 18 points, and they were categorized into three levels based on Bloom’s cut-off point of 60–80%, as follows: High level (80-100%) 15–18 points; Moderate level (60-79%) 11-14 points and a low level (less than 60%) 6–10 points [14].

##### Attitude toward antimicrobial usage and resistance

There were eight questions, one positive and seven negative statements, with Likert scale answers ranging from ”agree” to ”disagree.” A knowledge scale was used to assess the rating scale. The scores ranged from 8 to 24, and all individual responses were summed and calculated for means. The results were categorized into three (positive attitude, neutral attitude, and negative attitude). Positive attitudes ranged from 19–24 (80–100%), neutral attitudes ranged from 15–18 (60–79%), and negative attitudes ranged from 8–14 (less than 60%) [14].

##### Antimicrobial stewardship and resistance

This section contained eleven questions. There were ten closed-ended questions, eight Likert questions, one yes-or-no, one multiple-choice question, and one open-ended question. The previous rating scale was used to assess the responses. The scores in measuring antimicrobial usage and resistance ranged from 8 to 24 and were classified into three levels based on Bloom’s cut-off point of 60–80% [14]. The levels of practice were good (80–100%) of 19–24 scores, fair (60–79%) of 15–18 scores, and poor (less than 60%) of 8–14 scores [14].

### Data management and analysis

The collected data was classified, filtered, and coded using Microsoft Excel® 2010. The information was then exported to STATA version 16 (Stata Corp., Texas, USA) for statistical analysis. The socio-demographic characteristics of the study participants were shown using descriptive statistics. The numerical data were expressed as mean ± standard deviation or percentage when appropriate. The chi-square test (c2) was used to test the relationship between knowledge, attitude, and practice to identify the most important demographic factors. When the *p*-value is less than 0.05, the data is considered significant.

## Results

### Socio-demographic characteristics of respondents

The analysis of demographic parameters revealed that the vast majority of participants 301 (75.25%) were males, 128 (32%) were secondary school graduates, 294 (73.5%) were married, and 139 were employed in various government careers (34.75%). The average age of the study subjects was 43.85 years, with a standard deviation of 13.48 years (Table 1).

**Table 1:** Sociodemographic characteristics of study participants

**Variable**		**Number (*n*=400)**	**Percentage (%)**
**Sex**	Male	301	75.25
	Female	99	24.75
**Education level**	Primary	86	21.50
	Secondary	128	32
	Vocational	50	12.5
	College/university	71	17.75
	No formal education	65	16.25
**Marital Status**	Married	294	73.5
	Unmarried	89	22.25
	Divorced	9	2.25
	Widowed	8	2
**Occupation**	Government	139	34.75
	Nongovernment	68	17
	Student	45	11.25
	Private	94	23.5
	No work	54	13.5
**Household size**	Less than three	144	36
	Four to six	198	49.5
	Greater than six	58	14.5
**Animal ownership**	One species	176	44
	Two species	155	38.75
	Three or more species	69	17.25
Ownership of house	Private	157	39.25
	Rent	148	37
	Family	70	17.5
	Temporary shelter	15	3.75
	Other	10	2.5
**Position in the house**	Husband	212	53
	Wife	73	18.25
	Son	68	17
	Daughter	21	5.25
	Relatives	7	1.75
	Others	19	4.75

### Respondents’ knowing of antimicrobial use and resistance

The respondents’ mean knowledge score was 13.137 out of a possible 18 points (SD = 3.008). What’s more, approximately 48% of respondents had ”moderate knowledge,” 35% had ”high knowledge,” and 17% had ”low knowledge” about antimicrobial resistance and usage. Alternatively, as shown in Table 2, 54.5% of respondents were aware that antimicrobials were effective against bacteria. Similarly, half of the participants (51.5%) agreed that antimicrobials help with cold recovery. Approximately 47% disagree that antimicrobials are effective against the virus.

**Table 2:** Knowledge of respondents about antimicrobial usage and resistance

**Knowledge question**	**Agree**	**Uncertain**	**Disagree**	**Mean ± SD**
Do you think antimicrobials are effective against bacteria?	218	80	102	2.29± 0.85
	54.5	20	25.5	
Do you think antimicrobial speed up the recovery from the common cold?	206	94	100	2.26± 0.83
	51.5	23.5	25	
Do you think antimicrobials are effective against viruses?	112	101	187	1.81± 0.85
	28	25.25	46.75	
If you get adverse side effects during a course of antimicrobial treatment, do you stop taking antimicrobials?	225	85	90	2.33± 0.82
	56.25	21.25	22.5	
Do you think that the use of antimicrobials can increase the resistance of bacteria to them?	194	115	91	2.25± 0.80
	48.5	28.75	22.75	
Do you think that the use of antimicrobials in animals can reduce the effect of antimicrobials in humans?	172	126	102	2.17± 0.81
	43	31.5	25.5	

### Respondents’ attitudes toward antimicrobial use and resistance

More than half of the study participants (53.75%) were found to have a ”positive attitude,” 27.25% had a ”neutral attitude,” and 19% had a ”negative attitude” toward AMU and AMR. The mean attitude score for all respondents was 18.06 out of a possible 24 points (SD = 3.7).

According to Table 3, half of the respondents agreed with the statement ”to finish the course of treatment with AM even if they feel better.” About 35% of those polled agreed that they would seek antimicrobials from relatives or friends rather than from health care providers. However, more than half of the respondents (60.75%) disagreed with this statement. Similarly, the majority (74%) of respondents had a positive attitude (disagree) toward the statement, “*I prefer to be able to buy antimicrobials from the pharmacy without a prescription”*, and eighty-six (21.5%) of respondents agreed to do so, while nineteen (4.75%) remained uncertain. In addition, with the statement stated: ”*When I have a minor illness, I prefer to use an antimicrobial and feel better quickly,*” thirty-seven percent of respondents (148) agreed, 79 (19.75%) were unsure, and 173 (43.25%) disagreed (See Table 3).

**Table 3:** Attitude toward antimicrobial usage and resistance

**Attitude question**	**Agree**	**Uncertain**	**Disagree**	**Mean ± SD**
I always complete the course of treatment with antimicrobials even if I feel better	202	40	158	2.11± 0.94
	50.5	10	39.5	
It is good to be able to get antimicrobials from relatives or friends without having to see a medical doctor?	138	19	243	2.26± 0.94
	34.5	4.75	60.75	
I prefer to be able to buy antimicrobials from the pharmacy without a prescription.	86	19	295	2.52± 0.83
	21.5	4.75	73.75	
The effectiveness of antimicrobials is better if they are newer and more costly (A new brand than the common one and more expensive than the usual antimicrobials).	114	16	270	2.39± 0.90
	28.5	4.75	73.75	
Antimicrobials are safe drugs and can be commonly used	168	166	66	2.25± 0.72
	42	41	16.5	
I prefer to keep unused antimicrobials at home in case there may be a need for them	80	129	191	2.27± 0.78
	20	32.25	47.75	
When I have a minor illness, I prefer to use an antimicrobial and feel better quickly.	148	79	173	2.06± 0.89
	37	19.75	43.25	
Missing one or two doses does not alter the effectiveness of antimicrobials	131	65	204	2.18± 0.90
	32.75	16.25	51	

### Respondents’ antimicrobial use and resistance practices

All respondents had a mean practice score of 17.96 out of a possible 24 points (SD=2.73). Approximately half of the participants had a good practice, 39% had fair practice, and 11.25% had poor practice.

Two hundred seventy (54.25%) of those questioned said they always consult a doctor before beginning antimicrobial therapy. However, 42 (10.5%) of the participants took antimicrobials without consulting a health professional. Of the study participants, 61.25% always completed the full course of their antimicrobial treatment, 16.25% sometimes did, and 22.5% never finished. Furthermore, 60.5% of animal owners had never treated their animals with an antimicrobial prescribed for humans. However, 28% and 11.5% of respondents, respectively, treated their animals with antimicrobials prescribed for humans. Half of the respondents (50%) confirmed that they always complete the full course of treatment for their animals, 28.25% occasionally, and 21.75% never (Table 4).

**Table 4:** Public response to each practice question regarding antimicrobial usage and resistance

**Practice question**	**Always**	**Sometimes**	**Never**	**Mean ± SD**
Do you consult a doctor before starting an antimicrobial?	217	141	42	2.43± 0.68
	54.25	35.25	10.5	
Do you check the expiry date of the antimicrobial before using it?	240	99	61	2.44± 0.74
	60	24.75	15.25	
After you start feeling better, do you save the remaining antimicrobials for the next time you get sick?	60	175	165	2.26±0.70
	15	43.75	41.25	
After taking 2–3 doses and starting feeling better, do you give the leftover antimicrobials to your friend/roommate if they get sick?	84	117	199	2.28± 0.79
	21	29.25	49.75	
Do you complete the full course of treatment each time you take antimicrobials?	245	65	90	2.38± 0.83
	61.25	16.25	22.5	
Do you treat your animals with antimicrobials prescribed for humans by your decision?	46	112	242	2.49± 0.69
	11.5	28	60.5	
Do you take your animals to the Vet clinic for diagnosis?	79	215	106	1.93± 0.68
	19.75	53.75	26.5	
If you purchase antimicrobials for your animals, do you complete the full course?	200	113	87	1.71± 0.80
	50	28.25	21.75	

### Association between socio-demographic factors and respondents’ knowledge, attitude, and practice

Respondent knowledge level was significantly influenced by several socio-demographic characteristics, including education level (χ2 = 437.8, *p* < 0.01), marital status (χ2 = 22.49, *p* < 0.01), occupation (χ2 = 189.33, *p* < 0.01), house ownership (χ2 = 19.08, *p* = 0.01) and position in the house (χ2 = 28.44, *p* < 0.01) (Table 5). Similarly, participants’ attitude levels were significantly attributable to their education level (χ2 = 222.7, *p* < 0.01), marital status (χ2 = 20.53, *p* < 0.01), occupation (χ2 = 116.6, *p* < 0.01), and position in the house (χ2 = 22.95, *p* = 0.01) (Table 6). Furthermore, education level (χ2 = 178.6, *p* < 0.01) and occupation (χ2 = 111.5, *p* < 0.01) had a significant influence on the study participants’ practice level (Table 7).

**Table 5:** Association of knowledge with socio-demographic characteristics

**Variable**		**No of respondents**	**Knowledge**			**χ2**	***p-value***
			**High (%)**	**Moderate (%)**	**Low (%)**		
**Education level**	Primary	86	7 (8.2)	74 (86)	5 (5.8)	437.8	<0.01
	Secondary	128	44 (34.4)	82 (64)	2 (1.6)		
	Vocational	50	20 (40)	27 (54)	3 (6)		
	College/university	71	69 (97.2)	1 (1.4)	1 (1.4)		
	No formal education	65	0 (0.0)	7 (10.8)	58 (89.2)		
**Marital status**	Married	294	90 (30.6)	142 (48.3)	62 (21.1)	22.49	<0.01
	Unmarried	89	44 (49.4)	42 (47.2)	3 (3.4)		
	Divorced	9	3 (33.3)	5 (55.6)	1 (11.1)		
	Widowed	8	3 (37.5)	2 (25)	3 (37.5)		
**Sex**	Female	99	31 (31.3)	51 (51.5)	17 (17.2)	0.9	0.64
	Male	301	109 (36.2)	140 (46.5)	52 (17.3)		
**Occupation**	Government	139	92 (66.2)	43 (31)	4 (2.8)	189.3	<0.01
	Nongovernment	68	14 (20.6)	46 (67.6)	8 (11.8)		
	Student	45	12 (26.7)	32 (71.1)	1 (2.2)		
	Private	94	21 (22.3)	53 (56.4)	20 (21.3)		
	No work	54	1 (1.9)	17 (31.5)	36 (66.6)		
**Household size**	Less than three	144	48 (33.3)	71 (49.3)	25 (17.4)	1.75	0.78
	Four to six	198	72 (36.4)	95 (48)	31 (15.6)		
	Greater than six	58	20 (34.5)	25 (43.1)	13 (22.4)		
**Animal ownership**	One species	176	57 (32.4)	90 (51.1)	29 (16.5)	1.89	0.76
	Two species	155	58 (37.4)	71 (45.8)	26 (16.8)		
	Three or more species	69	25 (36.2)	30 (43.5)	14 (20.3)		
**Ownership of the house**	Private	157	57 (36.3)	69 (44)	31 (19.7)	19.08	0.01
	Rent	148	44 (29.7)	75 (50.7)	29 (19.6)		
	Family	70	32 (45.7)	36 (51.4)	2 (2.9)		
	Temporary	15	6 (40)	6 (40)	3 (20)		
	Other	10	1 (10)	5 (50)	4 (40)		
**Position in the house**	Husband	212	69 (32.5)	93 (43.9)	50 (23.6)	28.44	<0.01
	Wife	73	41 (56.2)	19 (26)	13 (17.8)		
	Son	68	33 (48.5)	33 (48.5)	2 (3)		
	Daughter	21	11 (52.4)	9 (42.9)	1 (4.7)		
	Relatives	7	4 (57.1)	3 (42.9)	0 (0.0)		
	Other	19	4 (21)	12 (63.2)	3 (15.8)

**Table 6:** Association of attitudes with demographic characteristics

**Variable**		**No of respondents**	**Attitude**			**χ2**	***p-value***
			**Positive (%)**	**Neutral (%)**	**Negative (%)**		
**Education level**	Primary	86	34 (39.5)	41 (47.7)	11 (12.8)	222.7	<0.01
	Secondary	128	82 (64.1)	42 (32.8)	4 (3.1)		
	Vocational	50	32 (64)	8 (16)	10 (20)		
	College/university	71	63 (88.7)	7 (9.9)	1 (1.4)		
	No formal education	65	4 (6.1)	11 (16.9)	50 (77)		
**Marital status**	Married	294	145 (49.3)	87 (29.6)	62 (21.1)	20.54	<0.01
	Unmarried	89	62 (69.7)	20 (22.5)	7 (7.8)		
	Divorced	9	4 (44.5)	2 (22.2)	3 (33.3)		
	Widowed	8	4 (50)	0 (0.0)	4 (50)		
**Sex**	Female	99	50 (50.5)	31 (31.3)	18 (18.2)	1.1	0.58
	Male	301	165 (54.8)	78 (25.9)	58 (19.3)		
**Occupation**	Government	139	107 (77)	20 (14.4)	12 (8.6)	116.6	<0.0
	Nongovernment	68	31 (45.6)	30 (44.1)	7 (10.3)		
	Student	45	26 (57.8)	14 (31.1)	5 (11.1)		
	Private	94	42 (44.7)	34 (36.2)	18 (19.1)		
	No work	54	9 (16.7)	11 (20.4)	34 (62.9)		
**Household size**	Less than three	144	68 (47.2)	47 (32.6)	29 (20.2)	9.1	0.06
	Four to six	198	115 (58)	52 (26.3)	31 (15.7)		
	Greater than six	58	32 (55.2)	10 (17.2)	16 (27.6)		
**Animal ownership**	One species	176	97 (55.1)	48 (27.3)	31 (17.6)	1.6	0.81
	Two species	155	79 (51)	42 (27.1)	34 (21.9)		
	Three or more species	69	39 (56.5)	19 (27.5)	11 (16)		
**Ownership of the house**	Private	157	86 (54.8)	39 (24.8)	32 (20.4)	8.7	0.37
	Rent	148	72 (48.6)	45 (30.4)	31 (21)		
	Family	70	44 (62.8)	19 (27.2)	7 (10)		
	Temporary	15	9 (60)	2 (13.3)	4 (26.7)		
	Other	10	4 (40)	4 (40)	2 (20)		
**Position in the house**	Husband	212	113 (53.3)	54 (25.5)	45 (21.2)	22.95	0.01
	Wife	73	33 (45.2)	24 (32.9)	16 (21.9)		
	Son	68	44 (64.7)	17 (25)	7 (10.3)		
	Daughter	21	15 (71.4)	6 (28.6)	0 (0.0)		
	Relatives	7	3 (42.9)	4 (57.1)	0 (0.0)		
	Other	19	7 (36.8)	4 (21.1)	8 (42.1)

**Table 7:** Association of practice with demographic characteristics

**Variable**		**No of respondents**	**Practice**			**χ2**	***p-value***
			**Good (%)**	**Fair (%)**	**Poor (%)**		
**Education level**	Primary	86	38 (44.2)	45 (52.3)	3 (3.5)	178.6	<0.01
	Secondary	128	69 (53.9)	58 (45.3)	1 (0.8)		
	Vocational	50	34 (68)	12 (24)	4 (8)		
	College/university	71	51 (71.8)	19 (26.8)	1 (1.4)		
	No formal education	65	7 (10.8)	22 (33.8)	36 (55.4)		
**Marital status**	Married	294	145 (49.3)	112 (38.1)	37 (12.6)	5.29	0.51
	Unmarried	89	46 (51.7)	38 (42.7)	5 (5.6)		
	Divorced	9	4 (44.5)	4 (44.5)	1 (11)		
	Widowed	8	4 (50)	2 (25)	2 (25)		
**Sex**	Female	99	48 (48.5)	41 (41.4)	10 (10.1)	0.39	0.82
	Male	301	151 (50.2)	115 (38.2)	35 (11.6)		
**Occupation**	Government	139	102 (73.4)	33 (23.7)	4 (2.9)	111.5	<0.01
	Nongovernment	68	33 (48.5)	31 (45.6)	4 (5.9)		
	Student	45	18 (40)	25 (55.6)	2 (4.4)		
	Private	94	39 (41.5)	44 (46.8)	11 (11.7)		
	No work	54	7 (13)	23 (42.6)	24 (44.4)		
**Household size**	Less than three	144	70 (48.6)	56 (38.9)	18 (12.5)	1.3	0.86
	Four to six	198	102 (51.5)	77 (38.9)	19 (9.6)		
	Greater than six	58	27 (46.6)	23 (39.7)	8 (13.7)		
**Animal ownership**	One species	176	88 (50)	67 (38.1)	21 (11.9)	2.12	0.71
	Two species	155	80 (51.6)	57 (36.8)	18 (11.6)		
	Three or more species	69	31 (44.9)	32 (46.4)	6 (8.7)		
**Ownership of the house**	Private	157	73 (46.5)	66 (42)	18 (11.5)	13.3	0.10
	Rent	148	77 (52)	49 (33.1)	22 (14.9)		
	Family	70	33 (47.2)	35 (50)	2 (2.8)		
	Temporary	15	10 (66.7)	3 (20)	2 (13.3)		
	Other	10	6 (60)	3 (30)	1 (10)		
**Position in the house**	Husband	212	106 (50)	75 (35.4)	31 (14.6)	10.5	0.40
	Wife	73	35 (50)	29 (39.7)	9 (12.3)		
	Son	68	33 (48.5)	32 (47.1)	3 (4.4)		
	Daughter	21	11 (52.4)	9 (42.9)	1 (4.7)		
	Relatives	7	5 (71.4)	2 (28.6)	0 (0.0)		
	Other	19	9 (47.4)	9 (47.4)	1 (5.2)		

### Relationship between respondents’ knowledge, attitude and practice

Respondent knowledge was found to have a significant relationship with respondents’attitudes toward AMR and AMU (χ2 = 215.23, *p* < 0.01). The proportion of respondents with positive attitudes rises as their level of knowledge rises (Sup Table 1). Similarly, a significant association (χ2 = 147.2, *p* < 0.01) was found between respondents’ knowledge and practice (Sup Table 2). It implied that, as the study participants’ level of knowledge of AMU and AMR increased, so did the proportion of respondents with good practice. Furthermore, a significant interaction (χ2 = 116.03, *p* < 0.01) was observed between respondents’ attitudes and good practices regarding AMU and AMR. It suggests that respondents’attitudes have a direct influence on their level of practice (Sup Table 3).

## Discussion

Inappropriate AMU and the associated risk of AMR are a growing public health issue worldwide [15–17]. The misuse and abuse of antimicrobials in agriculture, veterinary medicine, and human medicine have been identified as major contributors to the global spread of AMR [18–20]. The emergence and spread of antimicrobial-resistant pathogens impede the use of antibiotics for both preventative and therapeutic purposes. This issue is becoming more prevalent in low-income African countries [19, 21]. On the other hand, the use of antimicrobials can be affected by the interaction of knowledge and expectations of users and prescribers as well as the economic, health systems and environmental factors of the communities [16]. As a result, a questionnaire survey was used in this study to assess knowledge, practices, and attitudes toward AMU and AMR in Gondar City, Ethiopia. Knowledge, attitudes, and practices concerning antimicrobial use and resistance are critical for combating global antimicrobial resistance [22].

According to the findings of the current study, the majority of participants (72.3%) are aware of what antimicrobials are, but only 36.3% are conscious of what they are used for. Amoxicillin was the most commonly used antibiotic among the study participants. The current finding is consistent with the findings of Gebeyehu et al. [10] in Bahir Dar, Ethiopia; Widayati et al. [23] and Karuniawati et al. [24] in Indonesia; Sindato et al. [18] in Tanzania; Ocan et al. [25] in Uganda; and Ramay et al. [26] in Guatemala, all of whom found that Amoxicillin was the most commonly used antibiotic.

Understanding which conditions can be treated with antibiotics is also important, as using antibiotics for conditions that are not treatable with these medications contributes to misuse and, as a result, resistance development [23]. People in Gondar who participated in the study had sufficient knowledge (54.5%) to answer questions about whether antimicrobials are effective against bacteria, but this is lower than the previous studies in Germany [27] and Malaysia [28], which found that 83.7% and 76.7% of participants, respectively, correctly identified antibiotics as effective against bacteria. However, in the current study, some respondents (20%) are unsure whether antimicrobials are effective against bacteria, which is consistent with Kuwait (25.3%) [29]. Some attribute this lack of knowledge to the common use of the term ”germ” rather than the microbiological terms ”bacteria” or ”virus” during counselling or the provision of medical advice to the public or patients.

Poverty is also a major driver of AMR development in both developing and developed countries. In developing countries, factors such as insufficient access to effective drugs, unregulated antimicrobial dispensing and manufacture, and insufficient antimicrobial treatments due to cost contribute to the development of AMR [21]. According to a large number of participants (48.5%) in this study, the use of antimicrobials can increase bacterial resistance to them. This supports the findings of Tesfaye [30] in Bahir Dar, Ethiopia; Pereko et al. [31] in Namibia; Jifar and Ayele [32] in Harar, Ethiopia, and Darwish et al. [33] in Jordan, where 69.7%, 72%, 78.3%, and 50% of respondents believed, respectively. As a result, this finding indicates that the majority of those who took part in the study were well-versed in the risks associated with the use of antimicrobials. Antimicrobial agents are widely used in animal production systems in Ethiopia, as in other Sub-Saharan countries. On the other hand, the evidence on antimicrobial use is limited and anecdotal [34]. Antimicrobial resistance must be addressed through a variety of interventions, including interventions that reduce inappropriate and unnecessary antimicrobial use in humans and animals while also ensuring that effective antimicrobial therapy is available when needed [20, 35].

The effectiveness of antimicrobials is jeopardized by antimicrobial resistance, which can arise from discontinuing the entire course of treatment. According to studies, the reasons for the discontinuation of antimicrobials are a lack of knowledge about and awareness of antimicrobial use [10]. The participants’ attitude toward antimicrobial use and resistance was unavoidable and restrictive in this study. As a result, half of the respondents (50.5%) acknowledged the importance of continuing to take their prescribed medication even after they felt better. While approximately 40% of respondents believed that patients should discontinue treatment as soon as they felt better, this finding is higher than the study done in Bahir Dar, Ethiopia by Gebeyehu et al. [10], which reported 27%, and lower than the reports of Dyar et al. [35] in Kuwait, Sakr et al. [36] in Lebanon, and Darwish et al. [33] in Iraq, which reported that 45–60% of respondents, respectively, believed that patients should stop their treatment as soon as they felt better. Because of this misunderstanding in antimicrobial use, the patient is at risk of relapse with pathogenic bacteria resistance. Inadequate dosing, incomplete courses, and indiscriminate drug use have all contributed to the emergence and spread of antimicrobial resistance, which is now a problem in several countries [15].

Similarly, we practice self-medication every day in the form of self-care for our health. Self-medication refers to the use of drugs, herbs, or home remedies on one’s initiative or the advice of another person, without first consulting a doctor [37]. In this study, 54.25% of study participants had a positive attitude because they never took antimicrobials without first consulting with their doctor. However, 10.5% of them did not develop such an attitude and preferred to consult with and obtain antimicrobials from other sources. The current finding was lower than reports from other countries, including the UAE [38], Lebanon [39], Iraq [40], Palestine [41], Jordan [42], and Yemen [43]. Meanwhile, it was higher than reported by You et al. [44] in Hong Kong, McNulty et al. [45] in the United Kingdom, Ling Oh et al. [28] in Malaysia, Zajmi et al. [46] and Widayati et al. [23] in Indonesia, who reported attitude levels ranging from 4.8–9%. The observed differences in the studies were attributed to differences in sample size, education level, and sociodemographic characteristics of the study participants.

Furthermore, leftover antibiotics at home are major sources of medicine in many countries for emergency or future use issues [47]. In this study, 15% of respondents agreed that they keep leftover antimicrobials at home in case they need them in the future. The findings are comparable to Jifar and Ayele’s report in Harar, Ethiopia [32]. The current finding, however, was lower than that of a study conducted in Malaysia [48], Namibia [31], Jordan [33], India [17] and Egypt [49]. Disparities between studies could be attributed to differences in study participants’ awareness and education levels.

Moreover, significant associations (*p* < 0.05) between various socio-demographic factors and KAP scores were noticed in the current study. Antimicrobial knowledge scores tended to rise as one’s level of education increased. Similarly, participants with a college or university education, a secondary education, or a vocational education had higher knowledge scores than those with only primary education or no formal education. It corroborates the findings of Bhardwaj et al. [17] in India, Sindato et al. [18] in Tanzania, Geta and Kibret [20] in Ethiopia and Karuniawati et al. [24] in Indonesia. There was also a statistically significant (*p* < 0.05) relationship found between respondents’ knowledge and practice, knowledge and attitude, and practice and attitudes. As a result, it implied that as study participants’ knowledge of AMU and AMR increased, so did the proportion of respondents who practised and behaved well.

It is also necessary to describe the current study’s limitations. The information gathered was self-reported and based on the respondents’ memories, so it is subjective. The results could be underestimated or overestimated, resulting in recall bias. Furthermore, the quality of interview-based data may have been influenced by recall bias and the decision to share desirable and undesirable community practices. What’s more, the role of some relevant explanatory factors in our analysis was not ruled out. For example, household income status was not taken into account in this study. The study was also conducted in a small area with a snapshot approach, which may have resulted in selection bias because approximately 25% of the respondents were females. As a result, drawing generalizable conclusions from the results will be difficult. The findings, however, may apply to areas with similar settings.

## Conclusion

The current study’s findings indicate that there is some appropriate knowledge about antimicrobial usage and resistance. However, there were misunderstandings and a lack of knowledge about antimicrobial use, with respondents believing that antimicrobials were used to treat the common cold. Respondents demonstrated good practice, particularly in the use of antimicrobials, with the majority consulting a doctor before taking antimicrobials and refusing to take them from friends or pharmacies without a prescription. Meanwhile, there was a significant error in not taking the full dose of treatment. This malpractice reflected the public’s lack of knowledge and incorrect beliefs about the prudent use of antimicrobials. Respondents with a high educational level have good knowledge and understanding. This study’s findings are important because they provide valuable information for developing an intervention in public health promotion to improve knowledge, attitudes, and practices about antibiotics. To promote the prudent use of antibiotics, educational interventions on antibiotic use and its relationship with drug resistance are required, as is a Ministry of Health policy to increase knowledge and awareness about antibiotics, which includes mass media advertising.

## Supplementary Information

**Additional file 1: Table S1.** Association between knowledge and attitude level.

**Additional file 2: Table S2.** Association between knowledge and practice level.

**Additional file 3: Table S3.** Association between attitude and practice level.

## Data Availability

All data generated during this study are available to the authors upon request.

## Abbreviations

AMR: Antimicrobial resistance
AMU: Antimicrobial use
KAP: Knowledge, practice, and attitude
MDROs: Multidrug-resistant organisms

## References

[1] Abdallah EM (2011). Plants: An alternative source for antimicrobials. J Appl Pharm Sci.

[2] Drusano GL (2007). Pharmacokinetics and pharmacodynamics of antimicrobials. Clin Infect Dis.

[3] World Health Organization. Antimicrobial resistance: global report on surveillance. World Health Organization. 2014. https://apps.who.int/iris/handle/10665/112642.

[4] Dadgostar P (2019). Antimicrobial Resistance: Implications and Costs. Infect Drug Resist.

[5] Sahlan S, Wollny A, Brockmann S, Fuchs A, Altiner A (2008). Reducing unnecessary prescriptions of antibiotics for acute cough: adaptation of a leaflet aimed at Turkish immigrants in Germany. BMC Fam Pract.

[6] Moody J, Cosgrove SE, Olmsted R, Septimus E, Aureden K, Oriola S, Patel GW, Trivedi KK (2012). Antimicrobial stewardship: a collaborative partnership between infection preventionists and healthcare epidemiologists. Infect Control Hosp Epidemiol.

[7] Llor C, Bjerrum L (2014). Antimicrobial resistance: risk associated with antibiotic overuse and initiatives to reduce the problem. Ther Adv Drug Saf.

[8] Balala A, Huong TG, Fenwick SG (2019). Antibiotics resistance in Sub Saharan Africa; literature review from 2010–2017. Tanzania Vet J.

[9] Fasina FO, LeRoux-Pullen L, Smith P, Debusho LK, Shittu A, Jajere SM, Adebowale O, Odetokun I, Agbaje M, Fasina MM, Fasanmi OG, van Dyk D, Abubakar MS, Onakpa MM, Ali MG, Yousuf HS, Elmgboul WE, Sirdar MM (2020). Knowledge, Attitudes, and Perceptions Associated With Antimicrobial Stewardship Among Veterinary Students: A Multi-Country Survey From Nigeria, South Africa, and Sudan. Front Public Health.

[10] Gebeyehu E, Bantie L, Azage M (2015). Inappropriate Use of Antibiotics and Its Associated Factors among Urban and Rural Communities of Bahir Dar City Administration, Northwest Ethiopia. PLoS One.

[11] Erku DA, Mekuria AB, Belachew SA (2017). Inappropriate use of antibiotics among communities of Gondar town, Ethiopia: a threat to the development of antimicrobial resistance. Antimicrob Resist Infect Control.

[12] CSA (Central Statistical Agency). The Federal Democratic Republic of Ethiopia central statistical agency. Agricultural sample survey 2019/20 (2012 E.C.) (September-December 2019) volume III report on Farm Management Practices (Private peasant holdings, Meher season). Statistical Bulletin 446-514, Addis Ababa, 2020. https://www.statsethiopia.gov.et.

[13] Thrusfield M (2018). Veterinary epidemiology.

[14] Bloom BS (1956). Taxonomy of Educational Objectives.

[15] Al-Yasseri BJ, Hussain NA. Public knowledge and attitudes towards antibiotics use and resistance in Baghdad, Iraq: a survey conducted in the outpatient department of the university teaching hospital. Open Pub Health J. 2019;12(1):67-574. 10.2174/1874944501912010567.

[16] Alnasser AHA, Al-Tawfiq JA, Ahmed HAA, Alqithami SMH, Alhaddad ZMA, Rabiah ASM, Albrahim MAA, Al Kalif MSH, Barry M, Temsah MH, Al-Kalaif ZSH, Shahadah RFB, Alharbi KKS, Alnasser AAH (2021). Public knowledge, attitude and practice towards antibiotics use and antimicrobial resistance in Saudi Arabia: A web-based cross-sectional survey. J Public Health Res.

[17] Bhardwaj K, Shenoy S, Baliga S, Unnikrishnan B, Baliga BS (2021). Knowledge, attitude, and practices related to antibiotic use and resistance among the general public of coastal south Karnataka, India–A cross-sectional survey. Clin Epidem and Glob Hlth.

[18] Sindato C, Mboera LEG, Katale BZ, Frumence G, Kimera S, Clark TG, Legido-Quigley H, Mshana SE, Rweyemamu MM, Matee M (2020). Knowledge, attitudes and practices regarding antimicrobial use and resistance among communities of Ilala, Kilosa and Kibaha districts of Tanzania. Antimicrob Resist Infect Control.

[19] Geta K, Kibret M (2021). Knowledge, attitudes and practices of animal farm owners/workers on antibiotic use and resistance in Amhara region, northwestern Ethiopia. Sci Rep.

[20] Geta K, Kibret M (2022). Knowledge, Attitudes and Practices of Patients on Antibiotic Resistance and Use in Public Hospitals of Amhara Regional State, Northwestern Ethiopia: A Cross-Sectional Study. Infect Drug Resist.

[21] Samuels R, Qekwana DN, Oguttu JW, Odoi A (2021). Antibiotic prescription practices and attitudes towards the use of antimicrobials among veterinarians in the City of Tshwane, South Africa. Peer J.

[22] Bharti RK, Pathania JS, Sood V, Koshewara P, Dewangan T (2020). Assessing the Knowledge, Attitude, and Practice (KAP) of Antimicrobial Resistant among MBBS, BDS and BSc Nursing Students in the Northern State of India. An Observational-based Cross-sectional Study. Adv Biosci Clin Med.

[23] Widayati A, Suryawati S, de Crespigny C, Hiller JE (2011). Self-medication with antibiotics in Yogyakarta City Indonesia: a cross sectional population-based survey. BMC Res Notes.

[24] Karuniawati H, Hassali MAA, Suryawati S, Ismail WI, Taufik T, Hossain MS (2021). Assessment of Knowledge, Attitude, and Practice of Antibiotic Use among the Population of Boyolali, Indonesia: A Cross-Sectional Study. Int J Environ Res Public Health.

[25] Ocan M, Bwanga F, Bbosa GS, Bagenda D, Waako P, Ogwal-Okeng J, Obua C (2014). Patterns and predictors of self-medication in northern Uganda. PLoS One.

[26] Ramay BM, Lambour P, Cerón A (2015). Comparing antibiotic self-medication in two socio-economic groups in Guatemala City: a descriptive cross-sectional study. BMC Pharmacol Toxicol.

[27] Cals JW, Boumans D, Lardinois RJ, Gonzales R, Hopstaken RM, Butler CC, Dinant GJ (2007). Public beliefs on antibiotics and respiratory tract infections: an internet-based questionnaire study. Br J Gen Pract.

[28] Ling OhA, Hassali MA, Al-Haddad MS, Syed Sulaiman SA, Shafie AA, Awaisu A (2011). Public knowledge and attitudes towards antibiotic usage: a cross-sectional study among the general public in the state of Penang, Malaysia. J Infect Dev Ctries.

[29] Awad AI, Aboud EA (2015). Knowledge, attitude and practice towards antibiotic use among the public in Kuwait. PLoS One.

[30] Tesfaye Z (2017). Patient knowledge and practice on antimicrobial use and resistance in Felege Hiwot hospital, Bahir Dar. Ethiopia. J Basic Clin Pharma.

[31] Pereko DD, Lubbe MS, Essack SY (2015). Public knowledge, attitudes and behaviour towards antibiotic usage in Windhoek, Namibia. South Afr J Infect Dis.

[32] Jifar A, Ayele Y (2018). Assessment of Knowledge, Attitude, and Practice toward Antibiotic Use among Harar City and Its Surrounding Community, Eastern Ethiopia. Interdiscip Perspect Infect Dis.

[33] Darwish DA, Abdelmalek S, Abu Dayyih W, Hamadi S (2014). Awareness of antibiotic use and antimicrobial resistance in the Iraqi community in Jordan. J Infect Dev Ctries.

[34] Gemeda BA, Amenu K, Magnusson U, Dohoo I, Hallenberg GS, Alemayehu G, Desta H, Wieland B (2020). Antimicrobial Use in Extensive Smallholder Livestock Farming Systems in Ethiopia: Knowledge, Attitudes, and Practices of Livestock Keepers. Front Vet Sci.

[35] Dyar OJ, Yin J, Ding L, Wikander K, Zhang T, Sun C, Wang Y, Greko C, Sun Q, Stålsby Lundborg C (2018). Antibiotic use in people and pigs: a One Health survey of rural residents' knowledge, attitudes and practices in Shandong province. China. J Antimicrob Chemother.

[36] Sakr S, Ghaddar A, Hamam B, Sheet I (2020). Antibiotic use and resistance: an unprecedented assessment of university students' knowledge, attitude and practices (KAP) in Lebanon. BMC Public Health.

[37] Bennadi D (2013). Self-medication: A current challenge. J Basic Clin Pharm.

[38] Abasaeed A, Vlcek J, Abuelkhair M, Kubena A (2009). Self-medication with antibiotics by the community of Abu Dhabi Emirate, United Arab Emirates. J Infect Dev Ctries.

[39] Cheaito L, Azizi S, Saleh N, Salameh P (2014). Assessment of self-medication in population buying antibiotics in pharmacies: a pilot study from Beirut and its suburbs. Int J Public Health.

[40] Jassim AM (2010). In-home Drug Storage and Self-medication with Antimicrobial Drugs in Basrah, Iraq. Oman Med J.

[41] Al-Ramahi R (2013). Patterns and attitudes of self-medication practices and the possible role of community pharmacists in Palestine. Int J Clin Pharmacol Ther.

[42] Shehadeh M, Suaifan G, Darwish RM, Wazaify M, Zaru L, Alja'fari S (2012). Knowledge, attitudes and behaviour regarding antibiotics use and misuse among adults in the community of Jordan. A pilot study. Saudi Pharm J..

[43] Mohanna M (2010). Self-medication with Antibiotic in Children in Sana'a City, Yemen. Oman Med J.

[44] You JH, Yau B, Choi KC, Chau CT, Huang QR, Lee SS (2008). Public knowledge, attitudes and behaviour on antibiotic use: a telephone survey in Hong Kong. Infection.

[45] McNulty CA, Boyle P, Nichols T, Clappison P, Davey P (2007). The public's attitudes to and compliance with antibiotics. J Antimicrob Chemother.

[46] Zajmi D, Berisha M, Begolli I, Hoxha R, Mehmeti R, Mulliqi-Osmani G, Kurti A, Loku A, Raka L (2017). Public knowledge, attitudes and practices regarding antibiotic use in Kosovo. Pharm Pract (Granada).

[47] Tan YS, Hong CY, Chong PN, Tan ES, Lew YJ, Lin RT (2006). Knowledge that upper respiratory tract infection resolves on its own is associated with more appropriate health-seeking behaviour and antibiotic cognition. Singapore Med J..

[48] Lim KK, Teh CC (2012). A Cross-Sectional Study of Public Knowledge and Attitude towards Antibiotics in Putrajaya, Malaysia. South Med Rev.

[49] El Sherbiny NA, Ibrahim EH, Masoud M (2018). Assessment of knowledge, attitude and behaviour towards antibiotic use in primary health care patients in Fayoum Governorate, Egypt. Alexandria J Med.

