# Supplementary material for: Knowledge, attitude and practices of residents toward antimicrobial usage and resistance in Gondar, Northwest Ethiopia
Source: One Health Outlook. 2022 May 18;4:10. doi: 10.1186/s42522-022-00066-x (PMC9115959; doi:10.1186/s42522-022-00066-x)
Supplement: Supplementary file 1 — Additional file 1: Table S1. Association between knowledge and attitude level. [file 42522_2022_66_MOESM1_ESM.docx]

| **Attitude** | **Level** | **Knowledge** | | | | **χ2** | ***p*-value** | |  |
| --- | --- | --- | --- | --- | --- | --- | --- | --- | --- |
|  |  | **High** | **Moderate** | **Low** | **Total** |  | |  | |
|  |  | **N (%)** | **N (%)** | **N (%)** | **N (%)** |  |  |  |  |
|  | **Positive** | 121 (86.4) | 88 (46.1) | 6 (8.7) | 215 (53.7) | 215.23 | | 0.000 | |
|  | **Neutral** | 16 (11.4) | 80 (41.9) | 13 (18.8) | 109 (27.3) |  |  |  |  |
|  | **Negative** | 3 (2.2) | 23 (12) | 50 (72.5) | 76 (19) |  |  |  |  |
|  | **Total** | 140 (35) | 191 (47.8) | 69 (17.2) | 400 |  |  |  |  |
