# Supplementary material for: Knowledge, attitude and practices of residents toward antimicrobial usage and resistance in Gondar, Northwest Ethiopia
Source: One Health Outlook. 2022 May 18;4:10. doi: 10.1186/s42522-022-00066-x (PMC9115959; doi:10.1186/s42522-022-00066-x)
Supplement: Supplementary file 2 — Additional file 2: Table S2. Association between knowledge and practice level. [file 42522_2022_66_MOESM2_ESM.docx]

| **Practice** | **Level** | **Knowledge** | | | | **χ2** | ***p*-value** |
| --- | --- | --- | --- | --- | --- | --- | --- |
|  |  | **High** | **Moderate** | **Low** | **Total** |  |  |
|  |  | **N (%)** | **N (%)** | **N (%)** | **N (%)** |  |  |
|  | **Good** | 89 (63.6) | 96 (50.3) | 14 (20.3) | 199 (49.7) | 147.2 | 0.000 |
|  | **Fair** | 49 (35) | 88 (46.1) | 19 (27.5) | 156 (39) |  |  |
|  | **Poor** | 2 (1.4) | 7 (3.6) | 36 (52.2) | 45 (11.3) |  |  |
|  | **Total** | 140 (35) | 191 (47.8) | 69 (17.2) | 400 |  |  |
