# Supplementary material for: Knowledge, attitude and practices of residents toward antimicrobial usage and resistance in Gondar, Northwest Ethiopia
Source: One Health Outlook. 2022 May 18;4:10. doi: 10.1186/s42522-022-00066-x (PMC9115959; doi:10.1186/s42522-022-00066-x)
Supplement: Supplementary file 3 — Additional file 3: Table S3. Association between attitude and practice level. [file 42522_2022_66_MOESM3_ESM.docx]

| **Practice** | **Level** | **Attitude** | | | | **χ2** | ***p*-value** |
| --- | --- | --- | --- | --- | --- | --- | --- |
|  |  | **Positive** | **Neutral** | **Negative** | **Total** |  |  |
|  |  | **N (%)** | **N (%)** | **N (%)** | **N (%)** |  |  |
|  | **Good** | 132 (61.4) | 50 (45.9) | 17 (22.4) | 199 (49.7) | 116.03 | 0.000* |
|  | **Fair** | 80 (37.2) | 51 (46.8) | 25 (32.9) | 156 (39) |  |  |
|  | **Poor** | 3 (1.4) | 8 (7.3) | 34 (44.7) | 45 (11.3) |  |  |
|  | **Total** | 215 (53.7) | 109 (27.3) | 76 (19) | 400 |  |  |
